# P15 Reducing surgical site infections and antibiotic prescribing after Caesarean section with the use of dialkylcarbomoyl chloride coated (DACC) dressings

**DOI:** 10.1093/jacamr/dlae217.019

**Published:** 2025-01-23

**Authors:** Michael Magro, Tom Ashfield

**Affiliations:** Barking Havering & Redbridge University Hospitals NHS Trust (BHRUT), UK; Thesignpost, Winchester, UK

## Abstract

**Background:**

DACC wound dressings, in this case Leukomed^®^ Sorbact^®^, work by permanently binding bacteria, thereby reducing the bacterial load at the wound site during healing. Current rates of surgical site infections (SSI) after Caesarean section (CS) range from 3%–20%, however reporting is not mandatory in the UK and the true scale of the problem is unknown. A CS is the second most performed surgical procedure in the EU and increasing at an alarming rate. Inevitability, this will increase the occurrence of SSI, the associated antibiotic prescribing burden, healthcare costs and impact the lives of women, babies and their families in the post-natal period. Interventions that spare antibiotic use are vital as we endeavour to promote stewardship of antibiotic usage and respect the existential threat of AMR.

**Objectives:**

To assess if SSI rates could be reduced in women undergoing CS by using a DACC wound dressing (Leukomed^®^ Sorbact^®^) in a large outer London teaching hospital (circa 7000 births/annum).

**Methods:**

All women undergoing a CS from 1 January 2022–12 December 2022 (*n*=2368) had a DACC dressing applied to their wound and rates of SSI, readmission and antibiotic use were audited as part of a quality improvement project prospectively. Retrospective data from 1 January 2021–12 December 2021 was the comparison, during which an adhesive dressing (Mepore^®^) was the standard of care. No other changes were made to clinical practice during the study period and data were analysed using logistic regression.

**Results:**

There was a statistically significant reduction in SSI rates (OR 0.606, 95% CI 0.461–0.792, *P<*0.001, for women using a Leukomed^®^ Sorbact^®^ DACC wound dressing) from 2021 (SSI rate 6.1%, *n*=149) to 2022 (SSI rate 3.8%, *n*=91). This reduction remained statistically significant across all BMI categories and for both elective and emergency procedures. Rates of readmission, total readmission bed days and rates of antibiotic use for women readmitted with an SSI all fell by 30%, 38% and 30%, respectively. Total cost savings were £234 784 including a £49 750 saving for the Trust from reduced readmission costs.

**Conclusions:**

CS involves a transverse incision over the lower abdomen, which by its very nature sits under the abdominal pannus and is a moist/warm environment perfect for bacterial growth. By using Leukomed^®^ Sorbact^®^ dressings in all women undergoing CS, Barking Havering & Redbridge University Hospitals NHS Trust were able to significantly reduce SSI rates by 38%. This will have contributed to a significant reduction in antibiotics prescribed and more research should go into the use of DACC dressings as a way of improving antimicrobial stewardship. SSIs after CS causes wider effects than those reported above; including challenges with mother and baby bonding, breast feeding and mental health. Moreso, it is desirable to avoid antibiotic use in both mother and baby where possible. A significant challenge to improving SSI rates in the UK is the lack of standardized reporting across many units and hence SSI post caesarean, similar to orthopaedic procedures, should become a mandatory reporting target, thus allowing the scaling of projects to reduce SSI rates and antibiotic usage.

